# Comorbidities and Severe COVID-19 Outcomes: A Retrospective Analysis of Hospitalized Patients in Three Counties in Romania

**DOI:** 10.3390/microorganisms13040787

**Published:** 2025-03-29

**Authors:** Réka Bodea, Toader Septimiu Voidăzan, Lorand Iozsef Ferencz, Zoltán Ábrám

**Affiliations:** 1 Department of Epidemiology, George Emil Palade University of Medicine, Pharmacy, Science, and Technology of Targu Mureș, 540142 Târgu Mureș, Romania; septimiu.voidazan@umfst.ro; 2 Department of Hygiene, George Emil Palade University of Medicine, Pharmacy, Science, and Technology of Targu Mureș, 540142 Târgu Mureș, Romania; lorand.ferencz@umfst.ro (L.I.F.); zoltan.abram@umfst.ro (Z.Á.)

**Keywords:** COVID-19, comorbidity, retrospective studies, Romania, surveillance, odds ratio

## Abstract

The COVID-19 pandemic represents a major global health crisis, with clinical manifestations ranging from asymptomatic infection to fatal outcomes. While all individuals are susceptible, specific populations, particularly those with pre-existing medical conditions, face a heightened risk of severe disease. This study aimed to assess the prevalence of severe COVID-19 among hospitalized patients with comorbidities in the Central Region of Romania, and to analyze the association between these conditions and mortality. We conducted a retrospective cohort study using data from the Corona Forms platform (2020–2022), encompassing hospitalized cases across three Romanian counties. A total of 1458 patients with confirmed SARS-CoV-2 infection and documented comorbidities were included. Demographic characteristics, comorbid conditions, and hospitalization outcomes were analyzed. The overall mortality rate among comorbid patients was 89.3%. Renal, neurologic, hepatic disease, cardiovascular conditions, obesity, type 2 diabetes mellitus, and cerebrovascular accidents are significant risk factors for death outcomes in the SARS-CoV-2-infected study population. The strength of their association varies, with odds ratios ranging from 25.32 to 1. The findings underscore the critical impact of comorbidities on COVID-19 severity and mortality among the Central Romanian population, emphasizing the necessity of targeted clinical interventions and public health strategies to protect high-risk populations.

## 1. Introduction

Severe acute respiratory syndrome coronavirus-2 (SARS-CoV-2) was a major global health threat responsible for a prolonged and highly fatal pandemic, primarily causing respiratory disease with recurrent infection waves [1]. The clinical manifestations of COVID-19 range from asymptomatic infection to critical illness and mortality. Although SARS-CoV-2 infection poses a risk to all individuals, specific populations are disproportionately susceptible to severe disease. This susceptibility is influenced by factors such as age, gender, socioeconomic status, smoking history, and, most critically, underlying medical comorbidities [2,3,4,5,6,7,8]. Several pre-existing conditions have been identified as risk factors for severe COVID-19.

Chronic obstructive pulmonary disease (COPD) has been identified as a significant risk factor for both hospitalization and mortality in COVID-19, independent of age, sex, and other comorbidities. The increased mortality risk is attributed to pre-existing respiratory impairment, ventilation/perfusion mismatch, and secondary bacterial infections [9,10,11]. Respiratory support methods and barotrauma were also studied. Chest CT score and SpO_2_/FiO_2_ ratio contribute to worse clinical outcomes regardless of ventilatory strategies [12,13].

Metabolic syndrome. Several systematic reviews and meta-analyses have consistently demonstrated that metabolic comorbidities—including diabetes, obesity, dyslipidemia, and hypertension—synergistically exacerbate disease severity and mortality [14].

Diabetes mellitus (DM). Liu K. et al.’s meta-analysis highlights that DM significantly increases the risk of severe COVID-19 infection and mortality. Diabetic patients, particularly those with hypertension and other comorbidities, exhibit worse outcomes, including higher intensive care unit (ICU) admission and lower hospital discharge rates, due to exacerbated inflammatory responses, immune dysfunction, and increased susceptibility to complications such as cardiovascular and kidney diseases [15].

Obesity is a critical modifiable risk factor linked to increased inflammation, prothrombotic state, and impaired immune responses. Several pathophysiological mechanisms contribute to the heightened severity and mortality risk in obese individuals with COVID-19. Ectopic fat accumulation exacerbates COVID-19-induced inflammation. Additionally, obesity is associated with decreased levels of the anti-inflammatory adipokine adiponectin and overexpression of angiotensin-converting enzyme 2 (ACE2) receptors, potentially enhancing viral entry. Moreover, obesity contributes to endothelial dysfunction via multiple pathological pathways, including activation of the renin-angiotensin system, promotion of a procoagulant state, insulin resistance, oxidative stress, platelet dysfunction, and immune dysregulation [16,17,18,19].

Hypertension (HT). A Swedish study demonstrated that hypertension serves as an independent risk factor for severe COVID-19, regardless of age and other comorbidities. HT is associated with endothelial dysfunction, chronic inflammation, and immune dysregulation, potentially exacerbated by SARS-CoV-2-induced depletion of ACE2 [20,21,22,23,24].

Congestive heart failure. A South Korean population-based study demonstrated that a history of heart failure (HF) is significantly correlated with an increased risk of severe complications and mortality in COVID-19 patients [25]. The heightened mortality risk is due to pro-inflammatory cytokine release, myocardial injury, and thromboembolism [26,27,28].

Chronic kidney disease (CKD). A Swedish national study identified a strong and independent association between CKD and severe COVID-19 outcomes, including ICU admission and mortality, regardless of the underlying etiology of CKD. CKD is an independent predictor of severe COVID-19 and is characterized by immune dysfunction, cardiovascular risk, and predisposition to acute kidney injury [29,30,31].

Cancer. Both malignancy itself and anticancer therapies contribute to immune suppression, increasing the risk of infection and exacerbating the severity of SARS-CoV-2, particularly among patients with lung and hematologic cancer [32,33].

Thanks to public health surveillance and numerous clinical-epidemiological studies, several meta-analyses have been published post-pandemic, statistically supporting the role of multiple long-term conditions (MLTCs). The presence of two or more chronic diseases in an individual is associated with severe COVID-19 outcomes [34,35]. Some Romanian studies with smaller sample sizes have been conducted, accentuating the need for future policies in Romania to protect high-risk groups, provide personalized care, and offer guidance for healthcare policymakers to enhance clinical management [36,37,38,39]. This study aims to evaluate the association between various comorbidities and COVID-19 mortality in hospitalized patients, focusing on identifying the impact of specific comorbidities on the increased risk of death across different age groups in the Central Region of Romania.

## 2. Materials and Methods

### 2.1. Study Design

This retrospective cohort study was conducted in Romania’s Central Macroregion, as defined by the Eurostat Nomenclature of Territorial Units for Statistics (2024).

### 2.2. Data Collection

The study cohort included patients diagnosed with SARS-CoV-2 infection and recorded in the Corona Forms, a national database for tracking SARS-CoV-2 infections.

The dataset was obtained from the Corona Forms platform with authorization from the Public Health Directorate of each county. The Special Telecommunications Service developed Corona Forms at the request of the Ministry of Health to collect and integrate data from public authorities monitoring SARS-CoV-2 infections. The Ministry of Health, the National Institute of Public Health, county and municipal public health directorates, accredited RT-PCR testing laboratories, and healthcare facilities responsible for managing COVID-19 cases utilize it.

### 2.3. Sampling Method

The Central Macroregion comprises six counties: Alba, Brașov, Covasna, Harghita, Mureș, and Sibiu. A non-probability convenience sampling was applied. Only cases from three counties (Harghita, Covasna, and Mureș) were included.

Harghita, Covasna, and Mureș counties, according to the 2021 census, have a combined population of 2,271,067 inhabitants, representing 11.91% of Romania’s total population.

The inclusion criterion was isolation in public hospitals within the three selected counties between 26 February 2020, and 26 February 2022, covering the first two years of the COVID-19 pandemic in Romania.

In the studied counties, 15 public hospitals were involved in the isolation and management of COVID-19 patients, with a mandatory reporting obligation to the Corona Forms platform (see Appendix A).

### 2.4. Study Population

A total of 24,633 cases were identified and extracted from the Corona Forms database, excluding personal identifiers.

Following data collection, preprocessing was performed using Microsoft Excel. The study included only patients explicitly documented as having at least one comorbidity. Comorbidities were classified into the following 12 categories:

- Respiratory diseases (COPD, asthma, emphysema)
- Obesity (all grades)
- Type 2 diabetes mellitus (T2DM)
- Hypertension
- Chronic ischemic heart disease (IHD)
- Congestive heart failure (CHF)
- Atrial fibrillation (AF)
- Renal diseases
- Hepatic diseases (cirrhosis, steatosis, liver failure)
- Neurological disorders (excluding cerebrovascular accidents [CVA])
- Cerebrovascular accidents (CVA)
- Neoplastic diseases

A total of 1458 patients with documented comorbidities were identified.

Age was categorized into decades: <10, 11–20, 21–30, 31–40, 41–50, 51–60, 61–70, 71–80, and 91–99 years. The hospitalization period was determined based on the admission date, discharge date, or date of death.

### 2.5. Data Analysis

Quantitative data (e.g., age) were presented as means with standard deviation for normally distributed variables, while frequency tables were also used for categorical data. Qualitative data were summarized as counts and percentages. Statistical analysis was conducted using the Statistical Package for the Social Sciences (SPSS, version 23, Chicago, IL, USA). For categorical variables, the Chi-squared test was used to assess the association between comorbidities and mortality. The odds ratio (OR) was calculated to evaluate the strength of the association between comorbidities and the risk of mortality. A *p*-value of <0.05 was considered statistically significant for all tests.

## 3. Results

The number of cases recorded on the Corona Forms platform is summarized in Table 1. From these, only cases isolated in public hospitals in Mureș, Harghita, and Covasna counties were filtered and processed in our study (see Table 1).

**Figure 1:**
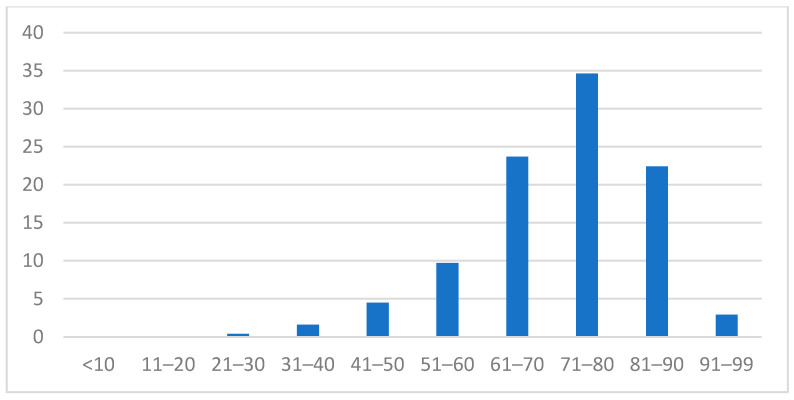
Characteristics of the study population—age distribution.

Out of 96,785 registered cases, only 24,633 (25.45%) required hospital isolation. Among hospitalized COVID-19 cases in Mureș, Harghita, and Covasna counties, during the first two years of the pandemic, 5.91% (1458) of cases had mentioned comorbidities. Males accounted for 52.7% of the analyzed cases. The mean age of the study population was 71.6 years (±12.7 SD), with the majority being elderly individuals over 60 years old (see Figure 1).

The median duration of hospitalization was 7 days (range: 0–393 days). The relationship between age and length of hospitalization (number of days) is presented in Table 2. (see Table 2).

**Figure 2:**
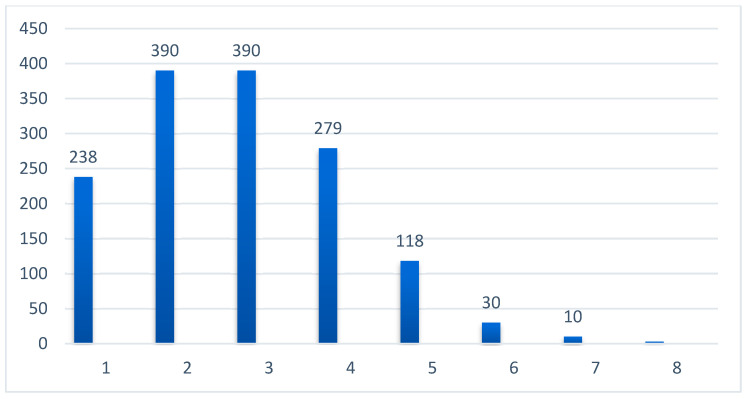
Incidence of number of associated comorbidities (numerical).

The distribution of comorbidities per patient is presented graphically in Figure 2. (see Figure 2). A total of 16.2% of patients had a single comorbidity, whereas those with more than seven comorbidities accounted for only 1.4% of the study sample.

The distribution of MLTCs by age group is shown in Table 3, illustrating that the number of comorbidities experienced by the studied population increases with age, especially in the 61–80 age range (see Table 3).

A total of 298 patients in the study cohort had renal comorbidities at the time of hospitalization for COVID-19. In contrast, 129 patients had a history of neoplastic disease, representing a relatively small proportion of the sample.

Among all comorbidities, ischemic heart disease (ICD) was the most frequent, affecting 988 patients. Regarding metabolic disorders, T2DM was more common than obesity, with 454 patients (31.13%) diagnosed with T2DM. Additionally, 102 patients (6.99%) had a history of stroke, representing the neurological sequelae within the cohort Table 4 (see Table 4).

The association between the number of comorbidities and mortality outcomes is presented in Table 5 (see Table 5).

To assess the correlation between the variable sets, a Chi-squared test was conducted, and the odds ratio (OR) was calculated. The results are presented in Table 6 (see Table 6).

In the present study, the contingency table data (see Table 6) revealed no statistically significant association between the presence of neoplastic comorbidities and mortality. Both the Chi-squared test and the calculated POR of 0.715 indicated no increased risk of death in patients with neoplastic comorbidities. The highest POR was observed between neurological comorbidities and mortality.

The analysis between the number of comorbidities and hospitalization duration in this dataset suggests no significant correlation. The correlation coefficient (ρ) was −0.019, indicating a very weak negative correlation between the two variables. This shows that an increase in the number of comorbidities is associated with a slight decrease in hospitalization duration; however, this relationship is negligible. The correlation was not statistically significant (*p* = 0.465, *p* > 0.05), indicating that the observed association could be due to chance rather than a true effect (see Table 7). This finding implies that the number of comorbidities alone may not be a determining factor in the length of hospital stay for patients.

## 4. Discussion

This study aimed to evaluate the association between multimorbidity and COVID-19 mortality in hospitalized patients, with a particular focus on the impact of specific comorbidities.

Multimorbidity, or MLTCs, refers to the coexistence of two or more chronic conditions and poses a considerable global public health challenge. The high prevalence of multimorbidity contributes to increased mortality, healthcare utilization, and associated costs. A systematic review and meta-analysis conducted by Chowdhury et al. assessed the prevalence and patterns of multimorbidity across various regions and over time. Between 2020 and 2021, the authors analyzed data from 126 peer-reviewed studies encompassing nearly 15.4 million individuals from 54 countries. The global prevalence of multimorbidity was found to be 37.2%, with more than half of the adult population over 60 years of age being affected by multimorbidity conditions [40].

The COVID-19 pandemic has further exacerbated health disparities, particularly for individuals with pre-existing conditions. While key risk factors for severe disease and mortality—such as older age, male sex, socioeconomic deprivation, and ethnic minority status—are well established, the impact of MLTCs on COVID-19 outcomes remains underexplored. Salisu-Olatunji et al. (2024) conducted a systematic review and meta-analysis to examine the influence of MLTCs on COVID-19-related morbidity and mortality. Their analysis, which included data from over 4 million individuals, revealed that MLTCs significantly increase the risk of severe COVID-19 outcomes and death. Patients with MLTCs were found to have more than twice the risk of mortality compared to those without MLTCs [35].

Additionally, Gebremedhn Gebremeskel et al. (2024) reported that the mortality rate among critically ill COVID-19 patients across 24 studies involving 142,291 participants ranged from 4.5% to 69.5%. Notably, 68.7% of these critically ill patients had chronic disease comorbidities [34]. In our study population of hospitalized COVID-19 patients, the mortality rate was 16.83%, and 89% in comorbid patients. The following section discusses the various comorbidities observed in our cohort, with a comparative analysis of the results reported in the existing literature.

Respiratory comorbidities. Meta-analyses involving 59 studies have consistently shown that COPD is associated with an increased risk of hospitalization, ICU admission, and mortality in COVID-19 patients [41]. A retrospective cohort study from the Republic of Korea also found COPD to be an independent risk factor for all-cause mortality, even after adjusting for age, sex, and the Charlson comorbidity index [42]. Similarly, a US study with over 11,000 COVID-19 patients identified COPD as the comorbidity most strongly associated with mortality, although obesity, diabetes, and hypertension were also independent predictors [43]. Moreover, a large cohort study from China involving over 39,000 patients revealed that patients with COPD and asthma had a higher likelihood of requiring invasive ventilation, ICU admission, or dying within 30 days of hospitalization after adjusting for age, sex, and other comorbidities [44]. In contrast, our study did not find a significant association between pulmonologic comorbidities and COVID-19 mortality. This could be due to several factors: the relatively small number of patients with pulmonologic conditions (*n* = 182), the inclusion of a wide range of pulmonary diseases (COPD, asthma, emphysema, etc.), varying severity levels, and inconsistencies in data collection across different centers.

Obesity. A large Swedish national study identified a strong, independent, and statistically significant correlation between obesity and severe COVID-19 outcomes, including mechanical ventilation, continuous renal replacement therapy, and death after ICU admission [29]. In 2022, a meta-analysis that included 3,140,413 COVID-19 patients from 167 studies found that obesity was associated with an increased risk of severe and high mortality [16]. Another meta-analysis from China, encompassing 46 studies, demonstrated that obese patients had significantly higher risks of hospitalization, severe illness, mechanical ventilation, ICU admission, and mortality [45]. Additionally, a Serbian study suggested that bioelectrical impedance analysis measurements could serve as more reliable predictors of severe obesity-related COVID-19 than body mass index alone [46]. Consistent with these findings, our study suggests that obesity may be associated with an increased risk of mortality from COVID-19 (OR = 1.002).

Type 2 diabetes mellitus. Sörling et al. identified a strong, independent, and statistically significant correlation between T2DM and severe COVID-19 outcomes, such as mechanical ventilation, continuous renal replacement therapy, and death after ICU admission [29]. Data from a meta-analysis underscore that, after adjusting for confounders, diabetes is a significant predictor of poor COVID-19 prognosis [4,5]. Our study suggests that T2DM may be associated with an increased risk of mortality from COVID-19 (OR = 1.458), although this association was not statistically confirmed.

High blood pressure. Research from 2022 indicated that hypertension alone was not an independent predictor of clinical outcomes, but was associated with poorer outcomes only when combined with diabetes or another risk factor [47]. It should be noted that some studies have shown no significant impact of hypertension or diabetes on the course of COVID-19, while others have reported that both hypertension and diabetes, with or without obesity, were independently linked to an unfavorable outcome [48]. Bauer et al. suggested that hypertension was an independent predictor of severe COVID-19 only in patients younger than 65 years, but not in the entire study population [49]. In contrast, Barrera et al., based on a study of 15,794 patients, reported that hypertension and diabetes, when considered separately, were significantly associated with ICU admission and death [50]. Thus, the impact of hypertension on the severity of COVID-19 remains controversial. In our study, we were able to demonstrate an association between hypertension and COVID-19 mortality, with a modest OR of 2.058, despite the ongoing debate on this topic.

Chronic ischemic heart disease. Our study shows a strong association between IHD and COVID-19 mortality (OR = 13.465), further emphasizing the severity of IHD as a comorbidity in COVID-19 patients. A meta-analysis encompassing 157,439 COVID-19 patients across 81 studies revealed that patients with pre-existing ischemic heart disease (IHD) faced an increased risk of mortality, severe/critical COVID-19, ICU admission, and a lower likelihood of discharge/recovery compared to COVID-19 patients without pre-existing IHD. In conclusion, pre-existing IHD significantly increases the risk of unfavorable outcomes in COVID-19 patients, particularly in male and hypertensive patients [51].

Congestive heart failure. A study conducted in Poland determined that patients hospitalized for COVID-19 with a history of congestive heart failure (CHF) had significantly higher in-hospital mortality (35% vs. 12%), and at three months (53% vs. 22%) and six months (72% vs. 47%) follow-up, compared to patients without a history of CHF. This study concluded that a history of CHF identifies patients with COVID-19 at high risk of in-hospital complications and mortality up to six months of follow-up [52]. Similarly, a national study from Korea found that the presence of CHF was independently associated with increased mortality [27]. Our study also suggests that CHF may be associated with an increased risk of mortality due to COVID-19 (OR = 2.524), although this association was not statistically significant (*p* > 0.05), likely due to the modest sample size.

Atrial fibrillation. A meta-analysis of 36 studies found pre-existing AF linked to higher in-hospital and post-discharge mortality, as well as increased ventilator use in COVID-19 patients [53]. However, a study of 31,000 U.S. patients showed that new-onset AF was not significantly associated with mortality, suggesting it may indicate disease severity rather than being an independent risk factor [54]. Our study found no link between AF and COVID-19 mortality, possibly due to the inability to distinguish between pre-existing and newly developed AF during hospitalization.

Renal comorbidities. Several studies have established that CKD increases the risk of severe COVID-19 outcomes, including hospitalization, mechanical ventilation, and mortality. A meta-analysis by Jdiaa et al. [31] highlighted a consistent association between CKD and COVID-19 severity, although the extent of its impact remains uncertain due to variations across studies. Similarly, other large studies report a significant correlation between CKD and poor COVID-19 prognosis, including a higher risk of requiring renal replacement therapy and increased mortality [29,30]. In line with these findings, our study confirms that renal comorbidities are strongly associated with COVID-19 mortality (OR = 3.677, *p* = 0.000), supporting the established role of CKD as a major risk factor for adverse COVID-19 outcomes. Investigations are needed to determine whether different stages of CKD influence COVID-19 mortality, and that will provide answers. A nationwide cohort study in Sweden also investigated the risk of severe COVID-19 associated with different CKD stages. The study found that worsening CKD stage and dialysis were independent risk factors for COVID-19-related hospitalization and mortality [55].

Hepatic comorbidities. A U.S. multicenter study identified alcoholic liver disease, cirrhosis, and hepatocellular carcinoma as risk factors for COVID-19 mortality [56]. Another study found cirrhosis linked to poorer 30-day survival [57], while a Korean analysis of non-alcoholic fatty liver disease (NAFLD) patients showed higher fatty liver index (FLI) scores correlated with severe COVID-19 outcomes [58]. However, our study found no link between liver comorbidities and COVID-19 mortality. This aligns with other studies suggesting that only severe liver conditions, such as cirrhosis and high FLI scores, affect COVID-19 outcomes. Future research should categorize liver comorbidities by severity for better clarity.

Neoplastic Comorbidities. Meta-analyses across diverse patient populations have shown that cancer patients with COVID-19 experience a mortality rate of approximately 22%, which is about four times higher than that observed in non-cancer patients. Subgroup analyses reveal even higher mortality in those with lung and hematologic cancers, with rates ranging from 33% to 34%, approximately double the rates seen in other cancer types. Additionally, severe outcomes, including ICU admission and mechanical ventilation, are consistently high, affecting 55% to 60% of cancer patients, regardless of cancer type [32,33]. In contrast, our study found no significant association between neoplastic comorbidities and COVID-19 mortality. This discrepancy may be attributed to the relatively small number of cancer patients in our cohort (n = 129), which could have limited our ability to detect a meaningful association. The impact of active (with the effect of cancer treatment) vs. remission cancer status on COVID-19 outcomes needs to be confirmed with further research.

Our findings raise the question of why certain comorbidities, such as chronic ischemic heart disease, have a stronger association with mortality than others, such as obesity. One possible explanation could be the underlying inflammatory processes or cardiovascular complications in these patients, which could exacerbate the effects of COVID-19. Future studies should explore these pathways in greater detail.

In addition to comorbidities, we also analyzed demographic parameters of the study population concerning COVID-19 mortality. Among the deceased, 63.44% were over 70 years of age, and 53.2% were male. However, while sex was not significantly associated with an increased risk of COVID-19 mortality, age demonstrated a clear correlation with mortality outcomes. A meta-analysis of 70 studies by Starke et al. found that the risk of in-hospital and case mortality increased by 5.7% and 7.4%, respectively, for each year of age, and the risk of hospitalization increased by 3.4% per year. The study did not identify a specific age threshold where the risk significantly accelerated, offering a quantifiable measure of how age influences COVID-19 severity [59]. Fabião et al. reported that men had a relative risk of 1.36 for mortality and 1.29 for COVID-19 severity compared to women. Importantly, age did not significantly influence the meta-regression results for either mortality or severity [60]. Our study, however, found no association between sex and an increased risk of COVID-19 mortality.

Study Limitations. The primary limitation of this study lies in the relatively small sample size (1458 patients), which increases the likelihood of a Type II error (β), whereby a statistically significant relationship that may exist is not detected due to insufficient statistical power.

Another limitation is the study design itself: this is a retrospective cohort study. This reliance on past data also introduces the potential for selection bias, which may reduce the generalizability and external validity of the findings. The retrospective nature of the study relies on previously collected data, which may be incomplete or inaccurate.

For instance, patients with chronic kidney disease (CKD) were grouped into a single heterogeneous category of renal comorbidities without differentiation by disease stage. Similarly, regarding obesity, there was a lack of information on obesity classes, which could have led to inconsistencies in the data analysis.

The observational nature of this study cannot establish a causal relationship; we can show associations and cannot definitively prove that one factor caused another.

## 5. Conclusions

This study highlights the strong impact of multimorbidity on COVID-19 mortality, with chronic ischemic heart disease, renal disease, and hypertension emerging as key risk factors. A novel finding is the exceptionally high mortality rate (89%) among comorbid patients, emphasizing the need for targeted interventions. Unlike prior studies, we found no significant link between neoplastic, respiratory, and liver comorbidities and mortality, likely due to sample size limitations. Our findings also raise questions about why some comorbidities, like chronic ischemic heart disease, pose greater risks than others, such as obesity, warranting further research. Despite limitations, this study underscores the need for personalized risk assessment in hospitalized COVID-19 patients.

## Figures and Tables

**Table 1:** Registered COVID-19 cases during the first two pandemic years in three central region counties of Romania.

	Mureș	Harghita	Covasna
26 February 2021.			
Confirmed cases	17,937	6239	5976
Reinfections	2385	571	689
Deaths	734	344	209
26 February 2022.			
Confirmed cases	58,040	20,548	18,197
Reinfections	4866	1180	1322
Deaths	1874	988	663

**Table 2:** Age analysis by decades and length of hospitalization in days.

Age Categories	Median	Min. No. of Days	Max. No. of Days	*p* Value
<10	41.00	3	79	
11–20	6.00	6	6	
21–30	5.00	1	11	
31–40	9.00	1	36	
41–50	8.00	0	26	
51–60	7.00	0	393	
61–70	8.00	0	58	
71–80	7.00	0	34	
81–90	7.00	0	35	
91–99	5.00	0	30	
Total	7.00	0	393	0.34

**Table 3:** Number of associated comorbidities divided into age groups.

	No. of Comorbities								
Age Categories	1	2	3	4	5	6	7	8	*p* Value
P < 10	1	1	0	0	0	0	0	0	
11–20	1	0	0	0	0	0	0	0	
21–30	4	1	1	0	0	0	0	0	
31–40	14	6	1	1	1	0	0	0	
41–50	26	21	7	9	1	1	0	0	
51–60	31	41	33	23	10	2	1	1	
61–70	60	89	94	59	31	8	2	2	
71–80	57	151	134	103	47	9	4	0	
81–90	39	69	109	76	23	8	3	0	
91–99	5	11	11	8	5	2	0	0	0.000

**Table 4:** Distribution of comorbidities by category.

Category of Associated Comorbidity	No. of Persons	%
Respiratory disease	182	12.48
Obesity	524	35.93
Type 2 diabetes	454	31.13
High blood pressure	903	61.93
Chronic ischemic heart disease	988	67.76
Congestive heart failure	145	9.94
Atrial fibrillation	172	11.79
Renal diseases	298	20.43
Hepatic disease	177	12.13
Neurological diseases	97	6.65
Cerebrovascular accident	102	6.99
Cancer	129	8.84

**Table 5:** Negative health outcomes and accumulation of associated comorbidities.

No. of Comorbidities/Person	Death		*p* Value
	Yes No (%) 1302 (89.3%)	No No (%) 156 (10.7%)	(Pearson Chi-Squared)
1	166 (12.7%)	72 (46.1%)	
2	337 (25.8%)	53 (33.9%)	
3	369 (28.3%)	21 (13.4%)	
4	273 (20.9%)	6 (3.8%)	
5	114 (8.7%)	4 (2.5%)	
6	30 (2.3%)	0 (0%)	
7	10 (0.7%)	0 (0.0%)	
8	3 (0.2%)	0 (0.0%)	0.000

**Table 6:** Multivariate analysis between age, sex, comorbidities, and death, OR value.

Variable	Death		*p* Value (Pearson Chi-Squared)	Odds Ratio; 95% CI
	Yes	No		
Sex				
Feminine	609 (46.8%)	81 (51.9%)	0.235	OR = 0.814; 95% CI = 0.584–1.135
Masculine	693 (53.2%)	75 (48.1%)		
Age				
<10	2 (0.2%)	0 (0%)	0.000	
11–20	1 (0.1%)	0 (0%)		
21–30	3 (0.2%)	3 (1.9%)		
31–40	13 (1.0%)	10 (6.4%)		
41–50	41 (3.1%)	24 (15.4%)		
51–60	108 (8.3%)	34 (21.8%)		
61–70	308 (23.7%)	37 (23.7%)		
71–80	471 (36.2%)	34 (21.8%)		
81–90	314 (24.1%)	13 (8.3%)		
91–99	41 (3.1%)	1 (0.6%)		
Comorbidities				
Respiratory diseases			0.366	OR = 0.804; 95% CI = 0.501–1.290
Yes	159 (12.2%)	23 (14.7%)		
No	1143 (87.8%)	133 (85.3%)		
Obesity			0.991	OR = 1.002; 95% CI = 0.709–1.417
Yes	468 (35.9%)	56 (35.9%)		
No	834 (64.1%)	100 (64.1%)		
Type 2 diabetes mellitus			0.053	OR = 1.458; 95% CI = 0.993–2.140
Yes	416 (32.0%)	38 (24.4%)		
No	886 (68.0%)	118 (75.6%)		
Hypertension			0.000	OR = 2.058; 95% CI = 1.473–2.876
Yes	831 (63.8%)	72 (46.2%)		
No	471 (36.2%)	84 (53.8%)		
Chronic ischemic heart disease			0.000	OR = 13.465; 95% CI = 8.734–20.757
Yes	961 (73.8%)	27 (17.3%)		
No	341 (26.2%)	129 (82.7%)		
Congestive heart failure			0.016	OR = 2.524; 95% CI = 1.159–5.496
Yes	138 (10.6%)	7 (4.5%)		
No	1164 (89.4%)	149 (95.5%)		
Atrial fibrillation			0.247	OR = 1.401; 95% CI = 0.789–2.486
Yes	158 (12.1%)	14 (9.0%)		
No	1144 (87.9%)	142 (91.0%)		
Renal diseases			0.000	OR = 3.677; 95% CI = 1.965–6.884
Yes	287 (21.8%)	11 (7.1%)		
No	1018 (78.2%)	145 (92.9%)		
Hepatic diseases			0.446	OR = 1.03; 95% CI = 0.46–2.28; *p* = 0.94
Yes	161 (12.4%)	16 (10.3%)		
No	1141 (87.6%)	140 (89.7%)		
Neurological diseases			0.000	OR = 25.31; 95% CI = 1.564–409.668
Yes	97 (7.5%)	0 (0%)		
No	1205 (92.5%)	156 (100%)		
Cerebrovascular accident			0.009	OR = 4.197; 95% CI = 1.315–13.400
Yes	99 (7.6%)	3 (1.9%)		
No	1203 (92.4%)	153 (98.1%)		
Cancer			0.210	OR = 0.715; 95% CI = 0.421–1.212
Yes	111 (8.5%)	18 (11.5%)		
No	1191 (91.5%)	138 (88.5%)		

**Table 7:** Results of a Spearman’s rank correlation analysis between the number of hospitalization days and the number of comorbidities.

			No. of Hospitalization Days	No. of Assoc. Comorb.
Spearman’s rho	No. of hospitalization days	Correlation Coefficient	1	−0.019
		Sig. (2-tailed)	.	0.465
		N	1443	1443
	No. of assoc. comorb.	Correlation Coefficient	−0.019	1
		Sig. (2-tailed)	0.465	.
		N	1443	1458

## Data Availability

The original contributions presented in this study are included in the article. Further inquiries can be directed to the corresponding author.

## Abbreviations

The following abbreviations are used in this manuscript:

ACE2	Angiotensin-converting enzyme 2
AF	Atrial fibrillation
BMI	Body Mass Index
CHF	Congestive Heart Failure
CKD	Chronic kidney disease
COPD	Chronic Obstructive Pulmonary Disease
CVA	Cerebrovascular Accident
DM	Diabetes Mellitus
FLI	Fatty liver index
HF	Heart Failure
HT	Hypertension
ICU	Intensive Care Unit
IHD	Chronic ischemic heart disease
MLTCs	Multiple long-term conditions
NAFLD	Nonalcoholic fatty liver disease
OR	Odds Ratio
SARS-CoV-2	Severe acute respiratory syndrome coronavirus-2
T2DM	Type 2 diabetes mellitus

## Appendix A

**County**	**Hospital Name**
Mureș County	Târgu Mureș County Emergency Hospital
(7 hospitals)	Emergency Institute for Cardiovascular Diseases and Transplantation, Târgu Mureș
	Mureș County Hospital
	Sighișoara Municipal Hospital
	Reghin Municipal Hospital “Dr. Eugen Nicoară”
	Târnăveni Municipal Hospital “Dr. Gheorghe Marinescu”
	Luduș City Hospital “Dr. Valer Russu”
Harghita County	Miercurea Ciuc County Emergency Hospital
(4 hospitals)	Odorheiu Secuiesc Municipal Hospital
	Gheorgheni Municipal Hospital
	Toplița Municipal Hospital
Covasna County (4 hospitals)	County Emergency Hospital “Dr. Fogolyán Kristóf”, Sfântu Gheorghe
	Târgu Secuiesc Municipal Hospital
	Baraolt City Hospital
	Cardiovascular Rehabilitation Hospital “Dr. Benedek Géza”, Covasna
